# A Single‐Center Retrospective Cohort Study of Biopterin Metabolism Disorders in the United Arab Emirates

**DOI:** 10.1002/jmd2.12468

**Published:** 2025-01-19

**Authors:** Omar Jarrah, Mahmood Nouri, Aisha Al Shamsi

**Affiliations:** ^1^ Pediatric Department Tawam Hospital Al Ain UAE; ^2^ Genetic and Metabolic Division, Pediatrics Department Tawam Hospital Al Ain UAE

**Keywords:** BH4, biopterin metabolism disorders, tetrahydrobiopterin deficiencies

## Abstract

**Background:**

Tetrahydrobiopterin (BH4) deficiencies comprise a group of five neurometabolic disorders caused by five genetic defects responsible for BH4 biosynthesis and regeneration. Their global prevalence remains unknown, and variance exists among different countries.

**Aims:**

To describe clinical, biochemical, molecular genetic data and follow‐up of patients with BH4 deficiency seen in Tawam Hospital.

**Methods:**

A retrospective study of all patients with BH4 disorders who were followed up between January 2010 and December 2023 in Tawam Hospital. All data were retrieved from patients' electronic charts, including baseline characteristics, developmental milestones, family history, and clinical examination. Radiological and laboratory investigations, including phenylalanine levels, prolactin levels, CSF study, urine and plasma pterin profiles, and molecular tests, were reviewed.

**Results:**

Ten patients with BH4 deficiencies were found. The youngest patient was diagnosed at 3‐ weeks, and the oldest was nine; 70% were male, consanguineous 80%, and 40% were Emirati. Prematurity was reported in two patients. Intrauterine growth restriction was found in 70%, and microcephaly in 40%. Eighty percent had developmental delay, and 2 patients had behavioral issues. Seizure and movement disorders were reported in five and three patients, respectively. Brain MRI showed cortical atrophic changes, corpus callosum hypoplasia, hyperintensities in the parieto‐occipital region, and periventricular white matter. Abnormal Newborn screening was found in 60%, with initial high phenylalanine levels (>120 μmol/L) in 80%. Prolactin level was high in all studied patients. Four patients' cerebrospinal fluid neurotransmitter metabolites were evaluated. Blood for DHPR screening and urine pterin profile were done on eight patients. Nine patients had molecular testing. DHPR deficiency was most commonly reported among studied patients (50%), with five novel variants in the *QDPR* gene. The second prevalent disorder was PTPS deficiency and only one patient with SR deficiency.

**Conclusion:**

This cohort offers an in‐depth clinical and genetic understanding of BH4 deficiencies from a single center in the UAE. It describes new genetic variants and addresses diagnostic challenges to enhance the patient's diagnosis and treatment.

## 1 Introduction

**FIGURE 1:**
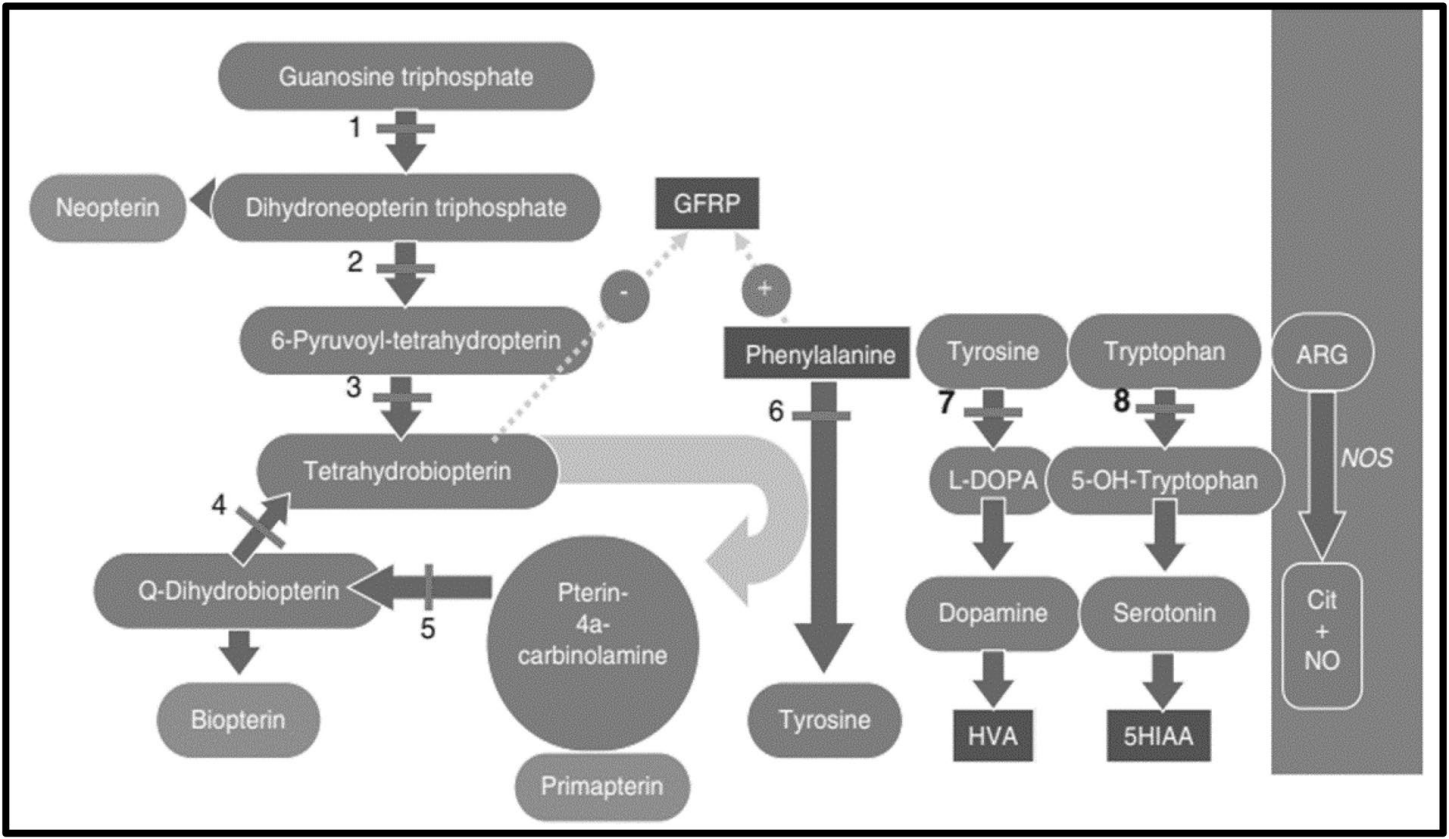
Tetrahydrobiopterin synthesis and deficiency states. Tetrahydrobiopterin synthesis and recirculation (left side) and enzyme reactions requiring BH 4 as a cofactor (right side). (1) GTP cyclohydrolase 1 (GTPCH); (2) 6‐pyruvoyltetrahydropterin synthase (PTPS); (3) sepiapterin reductase (SR); (4) dihydropteridine reductase (DHPR); (5) pterin 4alpha‐carbinolamine dehydratase (PCD); (6) phenylalanine hydroxylase (PAH); (7) tyrosine hydroxylase (TH); (8) tryptophan hydroxylase (TPH). This figure was copied from L.E. Bernstein et al. (eds.), Nutrition Management of Inherited Metabolic Diseases: 127 Lessons from Metabolic University, DOI 10.1007/978‐3‐319‐14 621‐8_12, Springer International Publishing Switzerland 2015. Chapter 12: Tetrahydrobiopterin Therapy for Phenylketonuria done by E. Jurecki.

Tetrahydrobiopterin (BH4) deficiencies comprise a group of five rare neurometabolic disorders caused by pathogenic variants in five genes responsible for the biosynthesis and regeneration of BH4. BH4 is needed for phenylalanine homeostasis, catecholamine synthesis, and serotonin biosynthesis. BH4 synthesis and regeneration involve a series of steps catalyzed by five enzymes. The enzymes used for BH4 biosynthesis are guanosine triphosphate cyclohydrolase 1 (GTPCH, EC3.5.4.16), 6‐pyruvoyltetrahydropterin synthase (6‐PTPS, EC 4.2.3.12), and sepiapterin reductase (SR, EC 4.1.1.17) causing guanosine triphosphate cyclohydrolase (GTPCH) deficiency (OMIM 233910), 6‐pyruvoyl tetrahydrobiopterin synthase (PTPS) deficiency (OMIM 261640) and sepiapterin reductase (SPR) deficiency (OMIM 612716), respectively. The enzymes used for BH4 regeneration are pterin‐4‐alpha‐carbinolamine dehydratase (PCD, EC 4.2.1.96) and dihydropteridine reductase (DHPR, EC 1.5.1.34) causing pterin‐4‐alpha‐carbinolamine dehydratase (PCD) deficiency (OMIM 264070) and dihydropteridine reductase (DHPR) deficiency (OMIM 261630), respectively [1, 2] (Figure 1).

All BH4 disorders are inherited in an autosomal recessive manner except GTPCH deficiency, which manifests in both autosomal recessive and autosomal dominant inheritance patterns.

Despite the differences in biochemical properties, similar clinical features are seen with variable severities in patients with BH4 deficiency. Poor sucking, hypotonia, hypertonia of the extremities, swallowing difficulties, myoclonic seizures, oculogyric crises, behavioral problems, sleep pattern disturbance, intellectual disability, dystonic movements, parkinsonism, temperature instability, and poor response to phenylalanine‐restricted diet, can be seen.

The global prevalence of BH4 deficiencies remains unknown, and significant variance exists among different countries [3, 4]. The mean incidence of hyperphenylalaninemia (HPA) detected by newborn screening (NBS) programs in Europe is estimated to be approximately 1:10000 [5], and BH4 deficiencies are presumed to constitute around 1–2% of these cases [6]. PTPS deficiency is the most frequent of all HPA‐associated BH4 deficiencies (approximately 54%), followed by DHPR deficiency (approximately 33%) [3, 6].

Looking into data about the United Arab Emirates (UAE), the reported incidence of classical phenylketonuria (PKU) diagnosed through a comprehensive national NBS program was 1:12369 in 2014 [7]. However, there are no published reports about BH4 deficiencies in the UAE.

It is worth knowing that a national NBS was established in the UAE in 1995 and used to be obtained between day 2 and day 4 after birth for all newborns. At first, it tested for PKU with a cutoff phenylalanine (Phe) level of <150 μmol/L. Disorders of amino acid, organic acid, fatty acids, and biotinidase were added in 2011 and became comprehensive in 2013 [7].

Emirati citizens have diverse ethnicities, including ancestors from the Arabian Peninsula, Persia, Baluchistan, and East Africa. The culture is tribal and favors intra‐tribal marriages. Related marriages are also common among most expatriates, mainly Palestinians and Pakistanis. The latest statistics showed 92 777 live births per year (citizens and non‐citizens) from all emirates in 2021 [8].

Our study aims to describe clinical, biochemical, and molecular genetic data and follow‐up of patients with BH4 deficiency due to various inherited genetic defects seen in Tawam Hospital.

## 2 Methods

This study was approved by the Abu Dhabi Health Research and Technology Ethics Committee (Ref. No.: DOH/CVDC/2023/2000). It is a descriptive retrospective study of all patients with BH4 disorders whose metabolic physicians had followed up between January 2010 and December 2023 in Tawam Hospital, UAE. All data were retrieved from the patient's electronic charts, including baseline characteristics, detailed history including developmental milestones, three‐generation family history, and thorough clinical examination. Radiological investigations such as brain MRI and laboratory investigations were reviewed, including Phe levels, prolactin levels, CSF study, urine and plasma pterin profiles, and molecular tests. Data was analyzed using Microsoft Excel 2022.

## 3 Results

Ten patients with BH4 deficiencies were found and included in this study. Molecular genetic tests were completed for nine patients; the family of one patient declined genetic testing.

Table 1 summarizes all the studied patients' clinical features and molecular results.

**TABLE 1:** Summary of all studied patients' clinical features and molecular results.

	Patient 1	Patient 2	Patient 3	Patient 4	Patient 5	Patient 6	Patient 7	Patient 8	Patient 9	Patient 10
**Ethnicity**	Emirati	Emirati	Omani	Indian	Indian	Emirati	Lebanese	Palestinian	Emirati	Palestinian
**Consanguinity**	2^nd^ cousins	No	1^st^ cousins	Distant relatives	No	1^st^ cousins	2^nd^ cousins	1^st^ cousins	1^st^ cousins	1^st^ cousins
**Gender**	Male	Male	Male	Male	Female	Female	Female	Male	Male	Male
**Age at diagnosis**	5 weeks	7 months	3 weeks	3 years	8 months	2 months	3 weeks	15 months	3 months	9 years
**Age at last** **follow‐up**	8 years	5 years	9 years	10 years	2 years	4 years	7 years	19 months	6 months	10 years
**GA at birth**	36 weeks	Term	Term	Term	38 weeks	33 weeks	39 weeks	37 weeks	31 weeks	Term
**HC at birth**	33 cm	36.5 cm	36 cm	Not available	Not available	27.3 cm	29 cm	30.5 cm	27 cm	34 cm
**Birth weight**	2.5 kg	3.3 kg	3.3 kg	4 kg	2.52 kg	1.31 kg	2.16 kg	2.08 kg	1.3 kg	2.77 cm
**Microcephaly**	No	No	No	Not available	No	Yes	Yes	Yes	Yes	No
**Seizure disorders**	No	No	Yes, at 9 years old	Yes, till 4 years old	At 7 months of age only	No	As neonate only.	Started at 3 months age	No	No
**Delay in Development**	No	Yes, mild weak hand grip, learning difficulty	No	Yes, moderate speech & motor delay	Yes	Yes, global delay	Yes, mild speech delay	Yes, global delay	Yes, moderate speech & motor delay	Yes, global delay
**Movement disorders**	No	Yes, ataxia	No	No	Yes, hands & tongue dystonia	No	No	No	No	Yes, ataxia
**EEG (if done)**	Not done	Not done	Not done	Left temporal epileptiform activity	Normal study	Not done	Normal study	Normal study	Not done	Not done
**Brain MRI** **(if done)**	Not done	Not done	Not done	Abnormal T2 hyperintensities in bilateral parieto‐occipital, & temporal lobe white matter (periventricular deep cortical subcortical ‘U’ fibers)	Cortical brain atrophic changes	Not done	Normal	Normal	Thin corpus callosum	Reduced white matter around occipital horns of lateral ventricles and intensity on T2WI periventricular white matter
**NBS Phe Result** ^**a**^	High Phe 339	High Phe 292	High Phe >1000	Not available	Reported as normal	High Phe 1659	High Phe 807	Reported as normal	1^st^ was normal, repeated Phe >1000	Reported as normal
**1** ^**st**^ **Phe level** **(μmol/L)**	579	512	517	Not available	516	1282	878	181	1501	50
**1** ^**st**^ **Tyr level (μmol/L)**	65	58	74	Not available	47	62	46	51	64	73
**Phe/Tyr ratio** ^b^	8.9	8.8	6.98	Not available	10.9	20.67	19	3.5	23.45	0.68
**Last Phe level** **(μmol/L)**	707	468	364	515	284	194	95	187	261	64
**1**st **Prolactin level (ng/mL)** ^c^	58.33	Not done	55.51	30.81	21.667	178.177	133.06	53.75	57.29	14.667
**Last Prolactin level (ng/mL)** ^**c**^	14.8	11.985	30.5	9.31	7.05	66.93	193.73	189.59	140.95	18.19
**DHPR blood** **(nmol/min/mg Hb)**	0 (normal > 0.7)	1.81 (normal 1.96–7.51)	0 (normal > 0.7)	Not done	0.24 (Normal: 1.96–7.51)	Biopterin was 0.02 mmol/mol Cr (normal 2.03–10.3), Neopterin total 11.57 mmol/mol Cr (normal 2.06–15.5). Biopterin is only 0.2% of sum of biopterin & neopterin. DHPR: 3.57 (normal 1.96–7.51)	1.3 nmol/min/mg Hb (Within Normal Limits > 0.7)	Reported as normal. The result is not available in the chart.	Blood biopterin was 0.01 mmol/mol Cr (normal 2.03–10.3), Neopterin total 13.2 mmol/mol Cr (normal 2.06–15.5). Biopterin is only 0.1% of sum of biopterin & neopterin. DHPR: 3.3 (normal 1.96–7.51)	Not done
**CSF study**	A CSF study was conducted after the treatment	Not done	Not done	A CSF study was conducted after the treatment started	Not done	Not done	A CSF study was conducted after 6 months of the treatment started	Not done	Not done	5‐HIAA: 7 nmol/L (normal 66–338) HVA: 93 nmol/L (normal 218–852), 3‐O‐methyldopa: 6 nmol/L (normal <100)
**Urine pterin profile**	30.4% Biopterin (normal: 11.5–50.7)	DHPR was significantly below the normal range	56.9% Biopterin (Normal: 11.5–50.7)	Not done	High level of biopterin, neopterin within reference range, with biopterin as 56% of sum of biopterin and neopterin	Significantly reduced level of biopterin and highly elevated level of neopterin	0% Biopterin (11.5–50.7)	Low level of biopterin, highly elevated neopterin with biopterin as only 3.6% of sum of biopterin and neopterin	0.1% Biopterin (11.5–50.7)	Not done
**Molecular confirmation**	Yes	Yes	Yes	Yes	Yes	Yes	Yes	Yes	No	Yes
**Variant**	Homozygous c.241G > T (p.Asp81Tyr) in *QDPR*	Heterozygous c.105 + 2 T > C & heterozygous c.122A > C (p.Asp41Ala) in *QDPR*	Homozygous c.49G > C (p.Gly17Arg) in *QDPR*	Homozygous c.156_158delCAT (p.152del) in *QDPR*	Homozygous c.630‐2A > G in *QDPR*	Homozygous c.315‐1G > A in *PTS*	Homozygous c.317C > T (p.Thr106Met) in *PTS*	Homozygous c.245A > G p.(Glu82Gly) in *PTS*	The diagnosis of PTPS Deficiency was made based on biochemical testing.	Homozygous c.560A > G (p.Glu187Gly) in *SP*
**Variant classification as per ACMG**	Predicted to be pathogenic	1^st^: likely pathogenic, 2^nd^: VUS	Pathogenic/likely pathogenic	Pathogenic	Likely pathogenic	Likely pathogenic	Likely pathogenic	Pathogenic/likely pathogenic	Not applicable	Likely pathogenic
**Treatment Provided** ^**d**^	Sapropterin, 5‐HT, L‐dopa (with carbidopa), Folinic acid	Sapropterin, 5‐HT, L‐dopa (with carbidopa), Folinic acid	Sapropterin, 5‐HT, L‐dopa‐carbidopa, Folinic acid	Sapropterin, 5‐HT, L‐dopa‐carbidopa, Folinic acid	Sapropterin, 5‐HT, L‐dopa‐carbidopa, Folinic acid	Sapropterin, 5‐HT, L‐dopa‐carbidopa, Folinic acid	Sapropterin, 5‐HT, L‐dopa‐carbidopa, Folinic acid	Sapropterin, 5‐HT, L‐dopa‐carbidopa, Folinic acid	Sapropterin, 5‐HT, L‐dopa‐carbidopa, Folinic acid	Levodopa‐carbidopa & Pramipexole
**Phe‐restricted diet**	No restriction	He was on a Phe‐restricted diet, but as there was no neurology, improvement was stopped	He was on a Phe‐restricted diet but stopped as it caused diarrhea. He is currently on a protein diet for preference of the family	Yes, Phe level was maintained below 250	He was on a Phe‐restricted diet, but as Phe levels were below 200, it was stopped	She was on a Phe‐restricted diet, but as Phe levels were below 200, it was stopped	She was on a Phe‐restricted diet, but as Phe levels were below 200, it was stopped	No restriction	He was on a Phe‐restricted diet, but as Phe levels were below 200, he had diarrhea and rash. It was stopped	No restriction
**Others**	None	Initial Pterin (urine & blood) cofactor tests showed elevated biopterin and neopterin, with biopterin as 45% of sum of biopterin & neopterin, which do not support BH4 disorders. DHPR was slightly reduced	Concern about hyperactivity and learning difficulty	Transferred care in 2020	Poor weight gain	Global developmental delay, hyperactive, autistic features	Tip toe walking	Hypotonia, failure to thrive	Congenital esophageal stricture, dysmorphic features, mild laryngomalacia, subtle collapse of arytenoids into glottis, hypotonia, duodenal perforation	Hypotonia, global developmental delay, learning difficulty

Abbreviations: 5‐HIAA, 5‐hydroxyindoleascetic acid; 5‐HT, 5‐hydroxytryptophan; ACMG, American College of Medical Genetics; CSF, cerebrospinal fluid; DHPR, Dihydropteridine reductase deficiency; EEG, electroencephalogram; GA, gestational age; HC, head circumference; HVA, homovanillic acid; MRI, Magnetic resonance imaging; NBS, newborn screening; Phe, phenylalanine; Tyr, tyrosine; VUS, Variant of uncertain significance.

^a^ The cutoff Phe level is < 150 μmol/L.

^b^ A normal Phe: Tyr ratio is typically < 1; a ratio of > 3 is considered useful in the diagnosis of PAH deficiency.

^c^ Prolactin's normal ranges are usually varied by age and sex.

^d^ Doses are based on book reference “Inborn Metabolic Diseases: Diagnosis and Treatment” while treating patients with BH4 deficiency.

### 3.1 Patients' Demographics

The youngest patient was diagnosed at 3‐ weeks of age (2 patients), while the oldest was nine years old. Seven (70%) were male, and 3 (30%) were female. Consanguinity was documented in 8 patients (80%) with no reported family history. Most of the patients studied were Emirati, 40%.

### 3.2 Clinical Phenotypes

This cohort identified five patients with DHPR deficiency, four with PTPS deficiency (one not molecularly confirmed), and one with SPR deficiency. No patients with GTPCH or PCD deficiency were identified. All patients with DHPR were born at term, while two patients with PTPS deficiency were born prematurely at 31 and 33 weeks of gestation. Their birth weights varied; the lowest was 1.3 kg, and the highest was 4 kg. Seventy percent of the patients had intrauterine growth restriction (IUGR). Microcephaly was documented in four patients (40%) who exhibited congenital microcephaly. Eighty percent of the patients had developmental delays, ranging from isolated speech to global delay (mild to moderate in severity). Behavioral problems, including hyperactivity and autistic features, were reported in two patients (one with DHPR deficiency and the other one with PTPS deficiency).

Seizure disorders were documented in 50% of patients with variable age of onset, starting as early as the neonatal period to 9 years of age. Four out of the five patients with seizures had an electroencephalogram (EEG) done, and only one showed epileptiform activity in the temporal region (unilateral), while the remaining were reported as normal studies.

Six patients underwent brain MRI studies for their developmental delay; two had no abnormalities, while the remaining four had variable findings, including cortical brain atrophic changes, corpus callosum hypoplasia, hyperintensities in the bilateral parieto‐occipital region, and periventricular white matter.

Movement disorders were reported in three patients; two had ataxic gait, and one had dystonic movements involving the tongue and hands.

One of our patients was found to have a congenital esophageal stricture, dysmorphic features, mild laryngomalacia, subtle collapse of arytenoids into glottis, hypotonia, and duodenal perforation, which was unclear if this was related to PTPS deficiency.

The reason those patients were brought to the metabolic clinic for evaluation varied between abnormal results of NBS for high Phe level (in 6 patients) or due to developmental delay (in 4 patients). Three of the studied patients had normal NBS results, which delayed the treatment, and one patient was diagnosed at three years old with no information about the NBS result.

Eight patients' initial Phe levels were high (>120 μmol/L) except for two; one with SPR deficiency had a level of 50 μmol/L (as expected), and one patient with DHPR deficiency was diagnosed abroad to have a high Phe level. All eight patients with high Phe levels had a high Phe: Tyr ratio > 3.

Prolactin level was higher than the reference range in all patients (considering prolactin's normal ranges usually vary by age and sex). Patients with PTPS deficiency had higher prolactin levels compared to patients with DHPR deficiency.

Four patients' cerebrospinal fluid (CSF) neurotransmitter metabolites were evaluated. Three had the study done after treatment was established, and one with SR deficiency had the CSF study done prior to treatment. The latter showed low levels of 5‐hydroxyindoleascetic acid (5‐HIAA), homovanillic acid (HVA), and 3‐O‐methyldopa. The other three patients who had the CSF study done after initiating therapy showed levels of 5‐HIAA and HVA within the reference ranges, suggesting adequate treatment.

Blood for DHPR screening was done on eight patients; 4 had low levels, as expected in patients with DHPR deficiency, and the remaining 4 had normal levels, as expected in patients with SR and PTPS deficiencies. The same eight patients also had a urine pterin profile, which showed extremely low biopterin in the four patients with PTPS deficiency.

### 3.3 Molecular Analysis

All nine patients who had molecular testing were confirmed to have either pathogenic, likely pathogenic, or predicted to be pathogenic variants. Two of the patients underwent whole exome sequencing, two others underwent the hyperphenylalaninemia panel, three had *QDPR* gene sequencing, and two had *PTS* gene sequencing. Most of the studied patients (50%) had DHPR deficiency (4 patients had homozygous variants, and one had compound heterozygous variants in the *QDPR*). The variants c.630‐2A > G, c.156_158delCAT (p.152del), c.105 + 2 T > C, c.122A > C (p.Asp41Ala), and c.241G > T (p.Asp81Tyr) in *QDPR* have not been previously reported (novel). The second prevalent disorder found in this study was PTPS deficiency, confirmed by molecular testing in 3 patients (with homozygous variants in the *PTS*) and by biochemical testing in one patient. Only one patient was confirmed to have a homozygous variant in the *SPR*, causing SR deficiency.

### 3.4 Response to Treatment

Seven patients were initially started on a Phe‐restricted diet due to a high Phe level, which was stopped after the diagnosis was established, or the Phe level dropped below 200 μmol/L, or the patient developed diarrhea or rash. One of these patients' Phe‐restricted diet was stopped as there was no neurological improvement. Another patient was continued on a Phe‐restricted diet upon family preference.

All nine patients were commenced on Sapropterin, L‐dopa (as a combined preparation with carbidopa), Hydroxytryptophan (5‐HT), and Folinic acid. The remaining patient with SR deficiency was kept on L‐dopa (as a combined preparation with carbidopa) and Pramipexole.

Patients with DHPR deficiency did relatively well clinically with their treatment regimen, although their Phe levels were not optimal. While patients with PTPS deficiency still had high Prolactin levels with some concerns in their developmental milestones and behavior.

## 4 Discussion

This cohort study provides essential information on the clinical, biochemical, and molecular genetic features of BH4 deficiencies among patients from the UAE for the first time. It emphasizes various critical factors concerning the epidemiology, clinical signs, diagnostic challenges, and genetic diversity of BH4 deficiencies. It also highlights the significant impact of these disorders, especially among populations with high consanguinity rates as in the UAE and Middle East.

Interestingly, in contrast to previously reported findings that PTPS deficiency was the most common subtype, we found DHPR deficiency is the most common among the studied patients here, with PTPS deficiency being the next.

The clinical manifestations of BH4 deficiencies vary widely, with symptoms ranging from mild to severe neurological deficits. Typical characteristics seen in the studied patients include developmental delay, seizures, unusual movements, IUGR, and microcephaly, which align with earlier research. Microcephaly, exhibited here as congenital, along with the IUGR, signifies the important prenatal developmental feature of PTPS deficiency. The patients with PTPS deficiency were noted to have a global developmental delay compared to those with DHPR deficiency, who had a milder delay.

The timing and seriousness of symptoms make it difficult to diagnose early, as some patients had normal/negative NBS results, leading to a delay in proper early intervention. This emphasizes the importance of increased clinical suspicion and thorough diagnostic assessment in infants and children with unexplained developmental delays or neurological symptoms. High prolactin and Phe levels can narrow the differential diagnoses in those children. As we found here, Prolactin levels are more pronounced in patients with PTPS deficiency than in patients with DHPR deficiency, which can also be used to monitor the treatment, with normal values indicating adequate L‐dopa replacement.

It is worth knowing that a high Phe:Tyr ratio does not constantly diagnose PAH deficiency, as 80% of patients in this cohort had a high ratio. This could mask and delay the diagnosis of BH4 deficiency.BH4 deficiencies can be diagnosed using biochemical assays (plasma and urine pterin) and molecular genetic testing. In this cohort study, genetic testing was conducted on all individuals except one patient, which showed variants in the *QDPR*, *PTS*, and *SPR* genes. Novel variants in the *QDPR* were found, thus highlighting the genetic variability in BH4 deficiency and the need for continued genetic monitoring to understand the correlation between genotype and phenotype and direct personalized therapeutic strategies.

Early diagnosis and prompt therapy are crucial for good prognostic outcomes. They can prevent permanent neurological damage and enhance the long‐term outlook. Treatment of BH4 deficiencies focuses on supplementing BH4, making dietary changes, and managing symptoms to improve neurological and developmental outcomes. The two available strategies to treat HPA are a Phe‐restricted diet or sapropterin dihydrochloride supplementation. No adverse effects of a Phe‐restricted diet have been documented; however, unnecessary dietary restrictions should be avoided, and daily Phe intake and tolerance should be closely monitored to optimize the maximal natural protein intake. The rationale for supplementing sapropterin dihydrochloride, a synthetic BH4 analogue, is based upon the defective biosynthesis or recycling of this essential cofactor for the aromatic L‐amino acid hydroxylases (AADC) in all types of BH4 deficiencies. The main effect of BH4 supplementation lies in its marked impact on controlling peripheral Phe levels [6]. BH4 deficiencies result in significantly reduced dopamine availability in the CNS. L‐Dopa, a dopamine precursor converted to dopamine by the AADC enzyme, has been widely used for numerous indications to restore dopamine homeostasis. Adding carbidopa blocks the decarboxylation of L‐Dopa, resulting in increased L‐Dopa concentrations at the blood–brain barrier and reduced peripheral L‐Dopa side effects [6].

This cohort backs the idea that early detection and treatment can significantly affect patient outcomes, emphasizing the significance of NBS initiatives and increasing knowledge among healthcare professionals.

The limitations of this study include the fact that data was collected from a single tertiary center, which contributed to the small sample size, and it is a retrospective study with the possibility of missing reported clinical features or missed follow‐ups. Therefore, further prospective multicenter, more extensive studies are recommended to describe BH4 deficiencies and their prevalence in the UAE properly.

## 5 Conclusion

In summary, this cohort offers an in‐depth clinical and genetic understanding of BH4 deficiencies among individuals seen in a single center in the UAE. It adds to the increasing knowledge about rare neurometabolic disorders by describing new genetic variants and addressing diagnostic challenges to enhance patient's diagnosis and treatment.

## Ethics Statement

All procedures followed were in accordance with the ethical standards of the responsible committee on human experimentation (institutional and national) and with the Helsinki Declaration of 1975, as revised in 2000 (5). This article contains no studies with human or animal subjects performed by the authors. This study was approved by the Abu Dhabi Health Research and Technology Ethics Committee (Ref. No.: DOH/CVDC/2023/2000).

## Conflicts of Interest

The authors declare no conflicts of interest.

## Data Availability

This manuscript has no associated data.
